# Estimated date of delivery with electronic medical records by a hybrid GBDT-GRU model

**DOI:** 10.1038/s41598-022-08664-5

**Published:** 2022-03-22

**Authors:** Yina Wu, Yichao Zhang, Xu Zou, Zhenming Yuan, Wensheng Hu, Sha Lu, Xiaoyan Sun, Yingfei Wu

**Affiliations:** 1 Engineering Research Center of Mobile Health Management Ministry of Education, Hangzhou Normal University, Hangzhou, China; 2 Hangzhou Hele Tech. Co, Hangzhou, China; 3 Hangzhou Women’s Hospital, Hangzhou, China

**Keywords:** Medical research, Experimental models of disease

## Abstract

An accurate estimated date of delivery (EDD) helps pregnant women make adequate preparations before delivery and avoid the panic of parturition. EDD is normally derived from some formulates or estimated by doctors based on last menstruation period and ultrasound examinations. This study attempted to combine antenatal examinations and electronic medical records to develop a hybrid model based on Gradient Boosting Decision Tree and Gated Recurrent Unit (GBDT-GRU). Besides exploring the features that affect the EDD, GBDT-GRU model obtained the results by dynamic prediction of different stages. The mean square error (MSE) and coefficient of determination (R^2^) were used to compare the performance among the different prediction methods. In addition, we evaluated predictive performances of different prediction models by comparing the proportion of pregnant women under the error of different days. Experimental results showed that the performance indexes of hybrid GBDT-GRU model outperformed other prediction methods because it focuses on analyzing the time-series predictors of pregnancy. The results of this study are helpful for the development of guidelines for clinical delivery treatments, as it can assist clinicians in making correct decisions during obstetric examinations.

## Introduction

Accurate estimated date of delivery (EDD) is helpful for pregnancy outcomes and clinical decisions making^1^, including diagnosing preterm and full-term, formulating measures for fetal dysplasia, arranging the timing of prenatal examination, preparing nursing measures for parturition and improving the efficiency of delivery. A reliable EDD is very important to reduce the occurrence of premature or postmature babies and is critical for both short-term and long-term health outcomes in neonates. Inaccurate EDD may have adverse effects on the health and safety of pregnant women and fetuses.

The current clinical method of determining the EDD is based on the information about last menstrual period (LMP) and ultrasound^2–4^. Among them, the Naegele’s rule based on LMP is the most common and wide method to calculate the EDD^5^. The Naegele’s rule is calculated by adding seven days and nine months to the first day of the LMP, or the EDD is 280 days after the first day of the LMP. However, the limitations of LMP include deviations in recalling the last menstruation, irregular menstrual cycles, oral contraceptives and early pregnancy bleeding^6^. In several studies, calculating EDD by ultrasound of the first trimester of pregnancy is more accurate than the LMP^7,8^. The research of Kessler et al.^9^ aimed at assessing the actual pregnancy length and accuracy of EDD prediction based on fetal head circumference measured at the second trimester, a population-based validation of 21,451 deliveries showed measurements can be safely used to predict EDD. Majola et al.^10^ compared the accuracy of LMP recall and an early ultrasound (EUS) in predicting the EDD in South African pregnant women, the results show that the effect of using EUS to calculate EDD is obviously better. However, the effect of predicting EDD only using ultrasonic features is not strongly helpful^11^. Interestingly, EDD is also affected by other factors. Obviously, pre pregnancy weight and maternal age are also important factors affecting EDD^12,13^. According to Staneva^14^, experiencing psychological distress such as depression, anxiety, and/or perceived stress during pregnancy may increase the risk of preterm birth. In addition, the life and behavior habits during pregnancy also affect EDD^15,16^. Besides, some studies believed that the accuracy of EDD will gradually decrease with the increase of gestational weeks^17^. Therefore, the EDD should be determined once we obtain the data from LMP or the first accurate ultrasound examination^18,19^. However, some studies showed that only about 5% of births are born exactly on EDD, regardless of the LMP methods or ultrasound^20^.

In the study of medical prediction methods, machine learning models are widely used since its high accuracy and high efficiency. Liang et al.^21^ used a linear regression model to find the blood metabolites that can predict gestational age and delivery date accurately. Heuvel et al.^22^ used convolution neural network (CNN) to estimate fetal head circumference, determine gestational age and delivery date. Fung et al.^23^ developed a machine learning approach based on ultrasound-derived and fetal biometric data to estimate gestational age and delivery date, but this article did not mention the type of ML. Schink et al.^24^ developed an algorithm to estimate the beginning of pregnancy in German claims data focusing on the potential of the expected delivery date. Torres et al.^25^ designed a system to calculate the gestational age and delivery date. They used images from the feet, face and ear of 130 newborn babies and a combination of fully convolutional networks, CNN and support vector regressors (SVR). Kojita et al.^26^ used fetal MRI in early pregnancy to predict gestational age and EDD. However, the above-mentioned prediction models ignored the effect of time series factors. Accurate EDD needs to evaluate the physical condition of pregnant women, and analyze the recent trend by judging the fetal development status of pregnant women at all stages. Since the data of antenatal examination is time series data, the EDD is closely related to the results of each examination.

Therefore, this study attempted to combine prenatal examination with electronic medical records to establish a hybrid time series model based on Gradient Boosting Decision Tree and Gated Recurrent Unit (GBDT–GRU) to predict the expected date of delivery.

## Methods

### Framework for the estimated date of delivery

**Figure 1:**
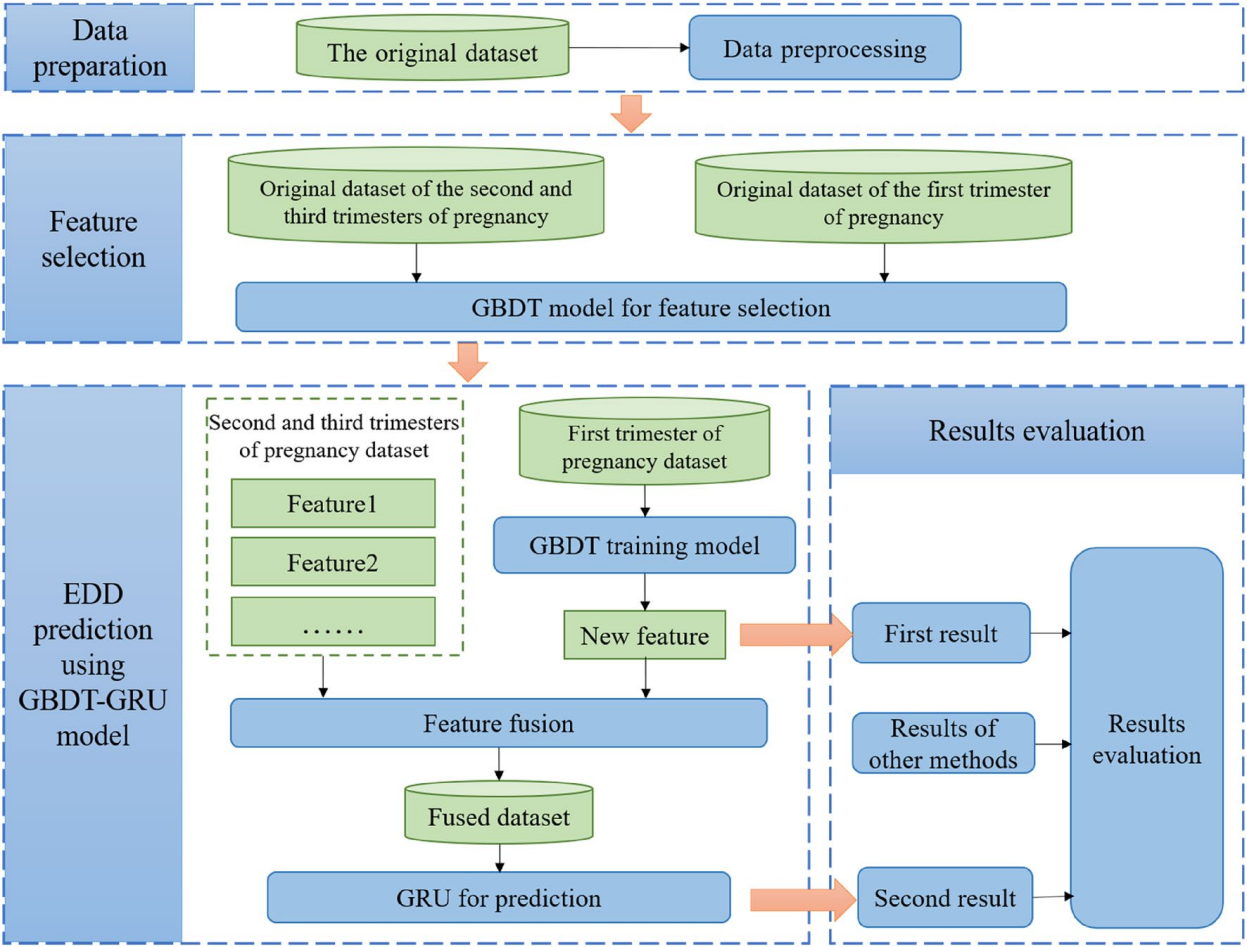
GBDT-GRU framework for the estimated date of delivery.

This study aimed to predict the EDD by using a hybrid model of GBDT and GRU. GBDT-GRU model made more effective and reasonable decisions by obtaining information from experience and mining hidden knowledge in data. The block diagram of the prediction process is shown in Fig. 1 and the detailed explanations of each step are as follows:

Step 1: Data preparation. The original maternal data obtained from EMR was processed with data cleaning and data transformation. Considering the different physiological characteristics during different periods of pregnancy, the processed data was divided into two datasets: dataset of the first trimester of pregnancy, and dataset of the second and third trimesters of pregnancy.

Step 2: Feature selection. Important features were selected in each dataset by ranking all feature importance of GBDT model, therefore avoiding the problem of information redundancy and reducing the dimension of data.

Step 3: EDD prediction using GBDT-GRU model. A preliminary prediction of EDD was achieved based on data of the first trimester with GBDT model, and considered as a new feature for the prediction of a more precise EDD. The new feature was combined with the original features of the second and third trimesters of pregnancy, and feed into a GRU model to generate the final EDD.

Step 4: Results evaluation. The prediction results of EDD with GBDT only, GBDT-GRU hybrid model and other methods were evaluated and compared.

### Datasets preparation

**Figure 2:**
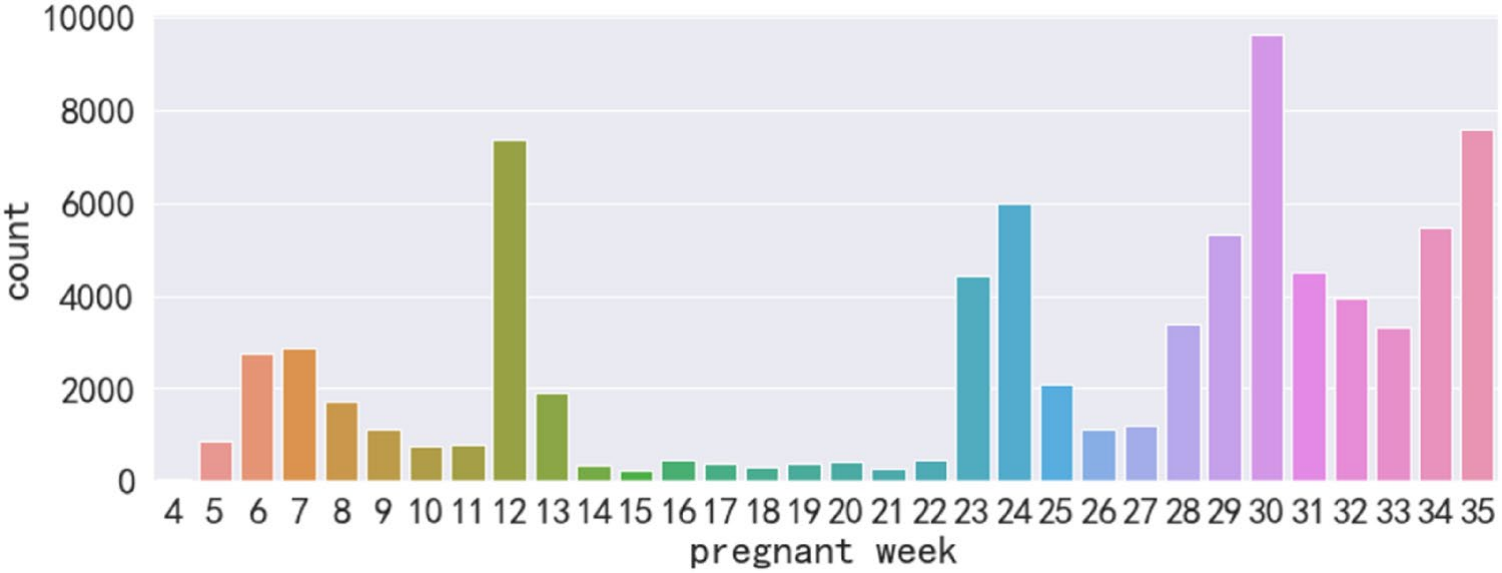
Count of the pregnant women in different pregnant weeks.

In this study, the data were collected from the electronic medical records (EMR) of pregnant women in a maternity hospital in eastern part of China. We extracted the physical examination and ultrasound records of the pregnant women who natural and full-term delivery between 2017 and 2020. The information was processed in such a way that the individual could not be identified. This study was approved by the ethics committee of Hangzhou Women’s Hospital and performed in accordance with the Declaration of Helsinki (written permission with approval NO. 2019-02-2). Considering the necessity of predict EDD in advance, only the physical examination records before 35 weeks were used in this study. The count of physical examinations of pregnant women in different gestational weeks is shown in Fig. 2. According to Fig. 2, there are too few pregnancy examinations between 13 and 22 weeks. In addition to the frequency of pregnancy examination, pregnant women have different examination items at different stages of pregnancy. Some ultrasound indicators only appear in the first trimester of pregnancy and will disappear with the increase of pregnancy weeks, such as the gestational sac (e. g, Features of the pregnancy examination Table 1). Therefore, we divided the dataset into two subsets according to the time of pregnancy examination: the first trimester of pregnancy dataset (pregnant week: 4 to less than 14 weeks); the second and third trimester of pregnancy dataset (pregnant week: 23 to less than or equal 35 weeks).

**Table 1:** Features of the pregnancy examination.

Types	Feature	Notation	Pregnant week
Static	Height	Height of pregnant woman (cm)	/
	Age	Age of pregnant woman	/
	P-W	Pre-pregnant weight of pregnant woman (kg)	/
	Gravidity	Gravidity	/
	Parity	Parity	/
	F-SBP	Systolic blood pressure of the first pregnancy examination	/
	F-DBP	Diastolic blood pressure of the first pregnancy examination	/
	LMP	Last menstrual period	/
	MD	Menstrual days	/
	MC	Menstrual cycle	/
	MA	Menarche age	/
	MV	Menstrual volume	/
	DY	Dysmenorrhea (0,1)	/
	DH	Disease history	/
Time series	SBP	Systolic blood pressure (mmHg)	4–35
	DBP	Diastolic blood pressure (mmHg)	4–35
	FUH	Fundal height (cm)	11–35
	AC	Abdomen circumference (cm)	11–35
	FHR	Fetal heart rate (times/min)	11–35
	HRF	High risk factors	4–35
	BMI	Body mass index (kg/m2)	4–35
	GSL	Gestational sac length (cm)	4–16
	GSW	Gestational sac width (cm)	4–16
	GSH	Gestational sac height (cm)	4–16
	FP	Fetal position	11–35
	PMG	Placental mature grading	12–35
	AFI	Amniotic fluid index (cm)	13–35
	S/D	Systolic to diastolic (ratio)	21–35
	NT	Nuchal translucency (cm)	10–14
	CRL	Crown-rump length (cm)	7–13
	BPD	Biparietal diameter (cm)	12–35
	FAC	Fetal abdomen circumference (cm)	12–35
	FL	Femur length (cm)	12–35
	HC	Head circumference (cm)	12–35
	HGB	Hemoglobin (g/L)	23–35
	BLG	Blood glucose (mmol/L)	23–35

Due to the variability and irregularity of pregnancy examination dates, some samples will be lost. In this work, we deleted the samples that lacked key features. For example, a sample only has basic features such as height and weight, but it lacks all important features such as gestational sac size, FAC, HC and so on. Moreover, samples with more than 50% missing values were excluded from further analysis. The antenatal examination data is time series data and linear interpolation is suitable for missing value filling of time series data. The missing values of our data were filled by linear interpolation according to the time of two adjacent pregnancy examinations. The gap between the values of variables, resulting from the different dimensions and dimensional units of variables, could affect the performance of the model. Therefore, it was necessary to normalize the data to avoid the influence of the larger range of values on other features and improve the convergence speed of the model. The min–max normalization is used to scale the values of the result to range [0,1], which is represented in Eq. (1) as:

1 $${x}^{*}=\frac{x-{x}_{min}}{{x}_{max}-{x}_{min}}$$

where $x$ is the current eigenvalue, ${x}_{min}$ and ${x}_{max}$ are the minimum and maximum values of the current feature, and ${x}^{*}$ is the standardized eigenvalue. The prediction results generated by the model need to be further denormalized as shown in Eq. (2), where $y$ is the true value and ${y}_{predict}$ is the predicted value.

2 $$y={y}_{predict}*\left({x}_{max}-{x}_{min}\right)+{x}_{min}$$

### Feature selection

GBDT^27^ is a boosting algorithm based on the classification and regression tree (CART). In this study, we used GBDT model for feature importance analysis and selection in the two datasets. The feature selection of GBDT is based on calculating the gain of the split nodes of the decision tree and using cumulative summation to evaluate the appropriateness of features^28^. The importance of a feature is measured by calculating the average importance of a feature in a single tree. GBDT uses formula (3) as a measure of influence, ${\widehat{I}}_{j}$ is the relative influence.

3 $${{\widehat{I}}_{j}}^{2}=\frac{1}{M}\sum_{m=1}^{M}{{\widehat{I}}_{j}}^{2}\left({T}_{m}\right)$$

where ${{\{T}_{m}\}}_{1}^{M}$ means a collection of decision tree, $M$ represents the number of trees. The importance of feature $j$ in a tree is calculated according to the formula (4):

4 $${{\widehat{I}}_{j}}^{2}\left(T\right)=\sum_{t=1}^{J-1}{{\widehat{i}}_{t}}^{2}1\left({v}_{t}=j\right)$$

where ${{\widehat{i}}_{t}}^{2}$ represents the squared loss, ${v}_{t}$ means a feature associated with $j$ nodes, and $J-1$ is the number of non-leaf node.

In the feature selection process, we generated the feature weights group ${{W=\{w}_{1} ,w}_{2},\dots {,w}_{n}\}$ from prenatal examination datasets and selection results of GBDT model, where ${w}_{i}$ describes the weight of each feature. Feature selection was performed on the two subsets based on the contribution degree of each feature. In this paper, we added the features one by one according to the weights from high to low, and selected the features used in this experiment by comparing the error and running time.

### Hybrid GBDT-GRU model

**Figure 3:**
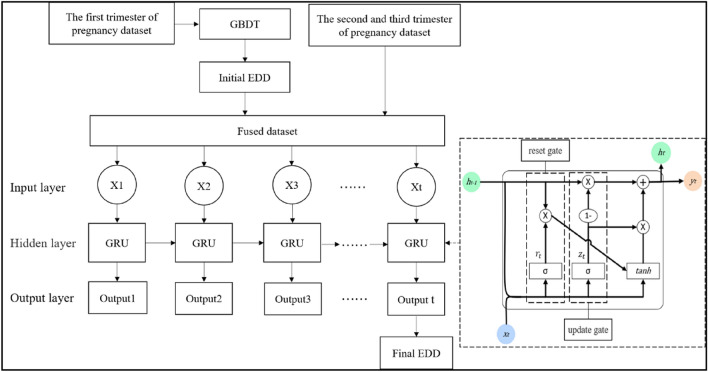
Structure of GBDT-GRU.

Since our aim was to predict the remaining days of pregnancy, the uncertainty of the future of pregnancy and the strict requirement of accuracy was really challenging. We designed a hybrid GBDT-GRU model and the structure was shown in Fig. 3. GBDT model is a kind of boosting algorithm, which belongs to the category of ensemble learning^29^. Among the machine learning methods used in practice, GBDT runs faster when training large amounts of data and have stronger robustness when processing outlier value. In this study, we used the GBDT model for the first prediction with the dataset of first trimester of pregnancy, then took the predicted results as the initial EDD. As a new feature, the initial EDD was fused with the second and third trimester of pregnancy dataset to obtain a fused dataset.

GRU^30^ and LSTM^31,32^ are variants of Recurrent Neural Network (RNN)^33^, they are proposed to solve the gradient disappearance and gradient explosion problems of traditional RNN in the process of long sequence training^34^. Different from LSTM, GRU only includes two gates: update gate and reset gate. The simplified structure enables GRU to effectively reduce the running time on the premise of ensuring the prediction accuracy. With the design of update gate and reset gate, GRU model can handle the time series data as well. The input layer of GRU is the time series from fused dataset, which can be noted as ${X=\{x}_{1}{,x}_{2},\dots , {x}_{t}\}$, where ${x}_{i}$ represents the record of the $i$th physical examination of pregnancy women. The hidden state ${h}_{t-1}$ contains the information of the previous node. Where ${z}_{t}$ and ${r}_{t}$ denote the update gate and reset gate, respectively. $Wr$ and $Wz$ are the weight matrices from hidden states at previous time step to the update gate and reset gate, respectively. $\sigma$ is a sigmoid function. The formula is expressed as follows:

5 $${r}_{t}=\sigma \left(Wr\cdot \left[{h}_{t-1 }{,x}_{t}\right]\right)$$

6 $${z}_{t}=\sigma \left(Wz\cdot \left[{h}_{t-1 }{,x}_{t}\right]\right)$$

The reset data obtained by the reset gate of the hidden layer data at the final moment is combined with the current input ${x}_{t}$, and $tanh$ is the activation function. The activation state of the hidden layer at the current moment ${\tilde{h }}_{t}$ can be defined as:

7 $${\tilde{h }}_{t}=tanh\left({W}_{\tilde{h }}\cdot \left[{r}_{t}*{h}_{t-1},{x}_{t}\right]\right)$$

Then the same gate ${z}_{t}$ is used to select and forget memory, and the hidden state ${h}_{t}$ of time $t$ can be calculated by:

8 $${h}_{t}=\left(1-{z}_{t}\right)*{h}_{t-1}+{z}_{t}*{\tilde{h }}_{t}$$

Finally, we used the result of the last moment of the output layer as the final EDD. Meanwhile, the final EDD could predict more accurately than initial EDD.

The parameters of these prediction models were determined by grid search and the models were validated with fivefold cross-validation. The grid search method combined all possible parameters, then trained each group of parameters to find the best combination of parameters. After five-fold cross-validation, the hyperparameter combination with the highest average score was taken as the best choice, and the model object was returned. The data used in the experiment included the records of multiple antenatal examinations of 5537 pregnant women, and dataset was divided into two sections, where 80% of dataset is used for training and the remaining 20% of dataset is used for testing. GRU model is composed of an input layer, a hidden layer and an output layer. During GRU training, the antenatal examination data are transformed into a three-dimensional matrix with a matrix size of (4430, X, Y), in which 4430 represents the number of pregnant women, X represents the number of antenatal examinations of pregnant women, and Y represents the number of characteristics of each antenatal examination of pregnant women. GRU model uses Adam optimizer to optimize the training process. This experiment is a regression task, so GBDT uses the mean square error “Ls” as the loss function of the algorithm. Table 2 shows the parameter settings of GBDT and GRU models.

**Table 2:** Parameters settings of GBDT and GRU models.

Model	Parameters	Values
GBDT	Learning rate	0.01
	Loss	“Ls”
	N_estimators	500
	Min_samples_leaf	4
	Min_samples_split	3
	Max_depth	3
GRU	Batch size	100
	Loss function	MSE
	Layers	1
	Optimizer	Adam
	Hidden_size	37
	Input size	18
	Learning rate	0.002
	Epochs	200

### Evaluation methodology

The prediction errors were considered as an essential factor to evaluate the proposed model. In this study, the coefficient of determination (R^2^), Mean Absolute Errors (MAE) and Mean Square Error (MSE) were used as the evaluation indices of the models. The calculation formulas are as follows:

9 $${R}^{2}=1-\frac{{\sum }_{i}^{n}{\left({\widehat{y}}^{\left(i\right)}-{y}^{\left(i\right)}\right)}^{2}}{{\sum }_{i}^{n}{\left(\overline{y }-{y}^{\left(i\right)}\right)}^{2}}$$

10 $$MAE=\frac{1}{m}\sum_{i=1}^{m}\left| {y}^{\left(i\right)}-{\widehat{y}}^{\left(i\right)}\right|$$

11 $$MSE=\frac{1}{m}\sum_{i=1}^{m}{({y}^{\left(i\right)}-{\widehat{y}}^{\left(i\right)})}^{2}$$

where ${y}^{(i)}$ and ${\widehat{y}}^{(i)}$ are the real and predicted values, respectively, and $\overline{y }$ is the average value of real values.

In order to further assess the effectiveness of prediction based on the GBDT-GRU model, the bias in predicting EDD of each method was used as another critical index of prediction reliability. The ${D}_{bias}$ is defined as formula (12), where ${D}_{real}$ is the actual date of delivery and ${D}_{predict}$ is the EDD.

12 $${D}_{bias}={|D}_{real}-{D}_{predict}|$$

By counting the proportion of people under different ${D}_{bias}$, we could get the performance and availability of different methods in practical applications. We calculated the accuracy under specific requirements ${Accuracy}_{bias}$ by formula (13).

13 $${Accuracy}_{bias}=\frac{{n}_{{D}_{bias}}}{N}*100\mathrm{\%}$$

where $N$ is the total number of pregnant women, ${n}_{{D}_{bias}}$ means the number of pregnant women whose prediction bias are less than or equal to ${D}_{bias}$.

### Ethics declarations

This study is observational and presents no more than minimal risk of harm to subjects and involves no procedures for which written consent is normally required outside the research context. The study was approved by the ethics committee of Hangzhou Women’s Hospital and performed in accordance with the Declaration of Helsinki (written permission with approval NO. 2019–02-2). The informed consent requirement for this study was waived by the ethics committee of Hangzhou Women’s Hospital. The researcher only accessed the database for analysis purposes, and all pregnant women data have been desensitized during the experiment.

## Results

### Description of the experimental data

The dataset used in this study comes from a hospital in the eastern part of China, which includes a large amount of data such as the maternal ultrasound records, prenatal examination reports and so on. After data preprocessing, the pregnancy dataset was obtained includes 33,222 pregnancy examination records and ultrasound records of 5537 pregnant women. Table 1 describes the features of the dataset.

### Feature importance and feature selection

**Figure 4:**
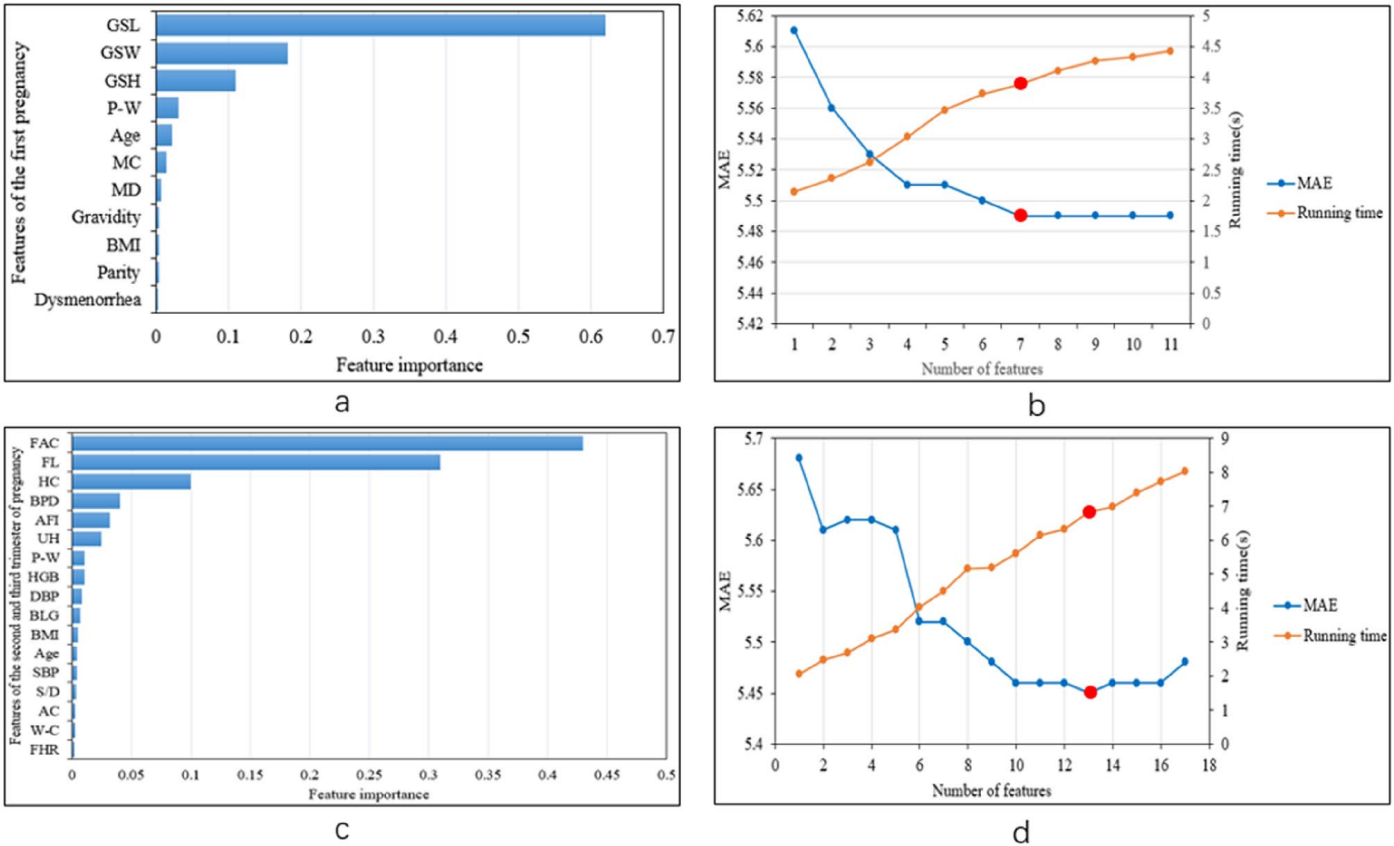
Analysis result for feature selection of different datasets. **a** shows the feature importance of the first pregnancy. **b** shows the MAE and running time with different number of features. **c** represents the feature importance of the second and third trimester of pregnancy. **d** represents the MAE and running time with different number of features in the second and third trimesters of pregnancy data.

We used GBDT to selected the features that have vital influences on EDD. The selected features were used as the input of the prediction model, which reduced the dimension of the input and solved the problem of information redundancy. Feature importance reflected the contribution of each variable in EDD. The results for feature selection of different datasets results are shown in Fig. 4a, c. The GSL was the most important variable to affect EDD in the first pregnancy dataset, followed by GSW, GSH, P-W, Age, MC and MD (Fig. 4a). At the same time, FAC was the feature with the highest weight value in the second and third trimester of pregnancy dataset, followed by FL, HC, BPD, AFI, UH, P-W, HGB, DBP, BLG, BMI, Age and SBP (Fig. 4c).

We added the features one by one according to the weights from high to low. The corresponding MAE values and running time after training the different number of features with GBDT are shown in Fig. 4b, d. We chose the feature group with the shortest running time in the case of the lowest MAE. Finally, seven features were retained in the first pregnancy dataset and 13 features were reserved in the second and third trimesters of pregnancy data. Table 3 shows the summary statistics of these parameters.

**Table 3:** Summary statistics of parameters.

First pregnancy	Value (Mean ± SD)	The second and third trimesters of pregnancy	Value (Mean ± SD)
Pregnant days	54.2 ± 10.8	Pregnant days	218.5 ± 14.9
GSL	3.6 ± 1.6	FAC	26.8 ± 2.4
GSW	2.7 ± 1.3	FL	5.8 ± 0.5
GSH	2.1 ± 1.2	HC	28.5 ± 1.8
P-W	53.9 ± 7.2	BPD	7.9 ± 0.6
Age	29.3 ± 3.4	AFI	11.7 ± 2.6
MC	30.6 ± 4.4	FUH	28.8 ± 2.5
MD	5.9 ± 1.1	P-W	53.5 ± 7.2
/	/	HGB	116 ± 10.1
/	/	DBP	67.3 ± 8.1
/	/	BLG	4.3 ± 0.4
/	/	BMI	24.4 ± 2.6
/	/	Age	29.3 ± 3.4
/	/	SBP	113.4 ± 10.7

### Evaluation and comparison of different models

In order to effectively evaluate the experimental results of GBDT-GRU model, we compared the prediction results of the Naegele’s rule and some machine learning models. The machine learning models we used for comparison include Random Forest (RF), Support Vector Regression (SVR) and LSTM. RF is a powerful algorithm for classification and regression, the prediction is made by majority vote or averaging the results of the ensemble^35,36^. SVR^37^ is an extension of the concept of Support Vector Machine (SVM), which is used for regression purpose. Based on the above-mentioned features in Table 3, we constructed these machine learning models to predict the EDD.

The average values of the results after fivefold cross-validation are shown in Table 4. We provided a performance comparison of the prediction models in different datasets. First, GBDT, RF and SVR were used to predict the initial EDD from the first trimester of pregnancy dataset. Second, the final EDD was gained with the time series model based on the fused dataset. Finally, MSE, R^2^ and training time were used to compare the prediction results of different models. Table 4 shows that the GBDT-GRU prediction model outperforms Naegele’s rule, all the single models and other hybrid models, achieves average MSE of 41.73 and R^2^ of 0.84. Moreover, comparing with the hybrid LSTM models, the hybrid GRU models have a shorter training time.

**Table 4:** Performance of different methods compared in two datasets.

Datasets	Method	MSE	R^2^	Training time (seconds)
First trimester of pregnancy dataset	Naegele’s rule	60.74	/	0
	RF	48.34 ± 0.2	0.61 ± 0.01	6.3
	SVR	48.66 ± 0.2	0.60 ± 0.01	620
	GBDT	46.73 ± 0.2	0.63 ± 0.01	3.9
	LSTM	47.35 ± 0.2	0.65 ± 0.01	436
	GRU	46.89 ± 0.2	0.65 ± 0.01	205
Fused dataset	SVM-LSTM	48.30 ± 0.2	0.80 ± 0.01	1025
	RF-LSTM	44.12 ± 0.2	0.81 ± 0.01	560
	GBDT-LSTM	46.13 ± 0.2	0.81 ± 0.01	510
	SVM-GRU	46.60 ± 0.2	0.82 ± 0.01	970
	RF-GRU	43.49 ± 0.2	0.83 ± 0.01	250
	GBDT-GRU	**41.73 ± 0.2**	**0.84 ± 0.01**	245

**Figure 5:**
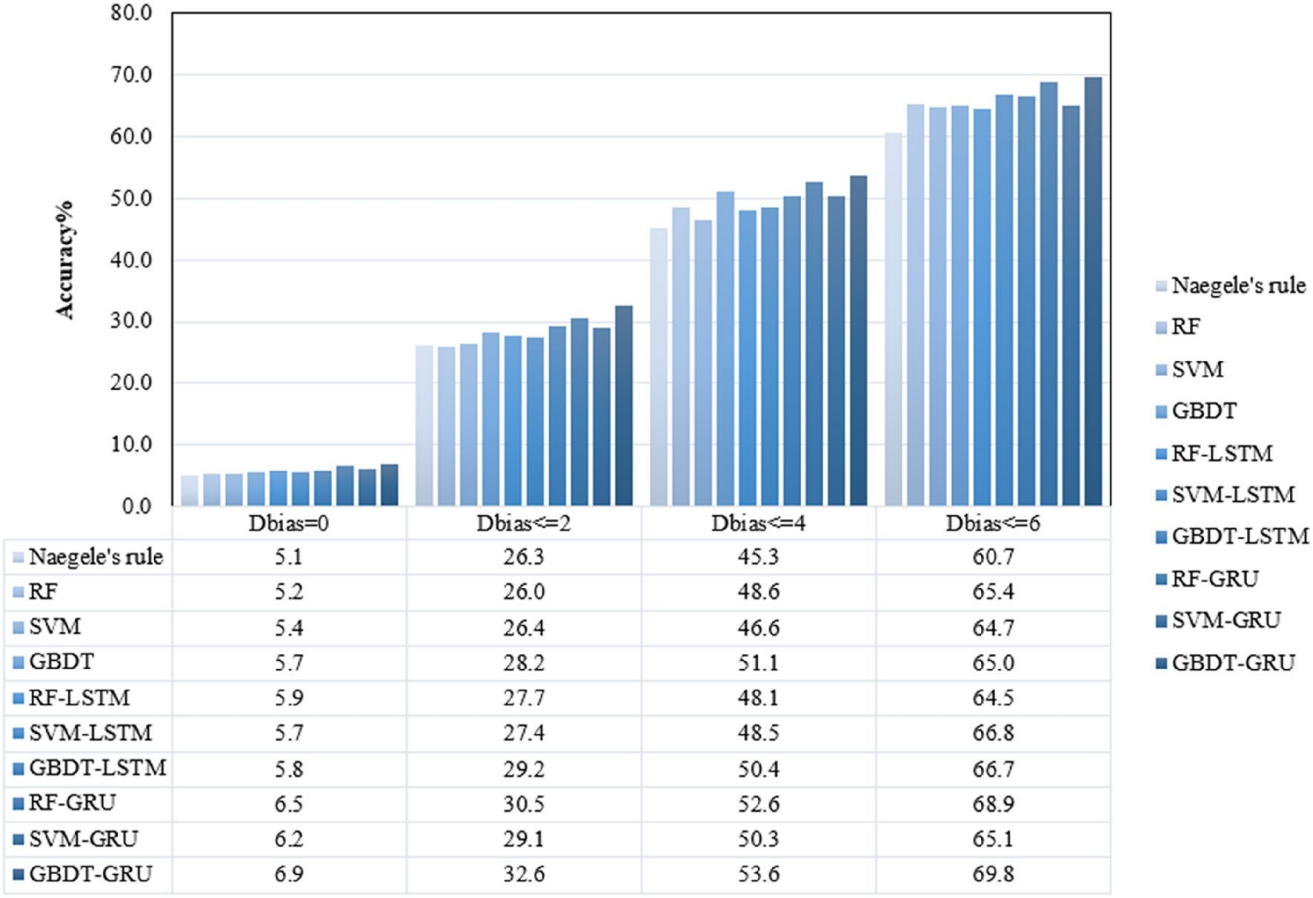
The accuracy of different methods under different ${D}_{bias}$.

According to the difference between EDD and the actual date of delivery, we recorded and compared the accuracy rate of each model under four categories: ${D}_{bias}$ smaller or equal to zero, two, four and six. The accuracy of different methods under different ${D}_{bias}$ is shown in Fig. 5.

As shown in Fig. 5, our GBDT-GRU models achieved better prediction results than other methods for different ${D}_{bias}$. The accuracy of EDD by GBDT-GRU model was 6.9%, 32.6%, 53.6% and 69.8%, when ${D}_{bias}$< = 0, 2, 4 and 6 days. Significantly, with the increase of ${D}_{bias}$, the accuracy advantage of GBDT-GRU model is more obvious.

## Discussion

In this study, we used a hybrid model of GBDT and GRU to generate features from EMR and to predicted the EDD of pregnant women. The accuracy of the GBDT-GRU model was superior to other prediction methods. In addition, we selected the features that have great influence on the EDD to make the model have better performance of prediction.

The experimental results showed that the performance of hybrid models (GBDT-GRU, GBDT-LSTM, RF-GRU and RF-LSTM) were better than all single models. Hybrid models achieved overall MSE is smaller than 44.12 and R^2^ is larger than 0.81. This shows that hybrid models have better generalization ability compared to other models for EDD, which may better serve and support the medical staff in decision making. Furthermore, the GRU presented better performance than LSTM when dealing with the time series data, which was benefited by the simpler gates structure of GRU. The GBDT-GRU exhibited the best performance among all models. As far as we know, this study was the first attempt to apply a hybrid model to the data of different stages of pregnancy, which could adjust the EDD according to the characteristics of each period of pregnancy. Therefore, it was obvious that our models were well suitable for the EDD of healthcare service.

As shown in Fig. 5, the proposed model not only optimizes the model running time but also improves the prediction accuracy. When ${D}_{bias}$ is less than six days, the accuracy of GBDT-GRU model is 9.1% higher than the Naegele’s rule. In addition, the results of this study were helpful for the EDD and had development of guidelines for clinical delivery treatments.

The clinical research about EDD was still focused on ultrasound and LMP, such as head circumference, cervical length, some improved formula methods^38^ and so on. These studies provided a reference for feature selection of machine learning. In addition, datasets of EMR provided great potential for EDD in pregnancy. We found that several new features were closely related to childbirth, which could enhance the accuracy of the EDD. The results of our study indicated that days of pregnancy, gestational sac size have a great influence on EDD in the first trimester of pregnancy. And for the second and third trimester of pregnancy, the influence of days of pregnancy, FAC, AFI and BPD were relative important features. Moreover, the importance of features given by GBDT model provides a reference for doctors to pay more attention to the key physiological indicators of pregnant women.

Our study also had several limitations that need to be improved. First, this study only used physical examination data and ultrasound data for prediction. We did not consider the influence of laboratory parameters on EDD. Second, the primary limitation of our study was a possible selection bias due to the center study with small sample size, and its accuracy and practicality should be verified in prospective studies with larger samples.

## Conclusions

In this paper, a hybrid model of a GBDT model and GRU model was proposed to predict EDD. For a more accurate EDD, we established a hybrid model of the parameters related to pregnant women and fetal physical examination. The results show that GBDT-GRU achieves a satisfactory outcome in the experiment and the accuracy of the EDD can be improved by adjusting the number of features. Therefore, our hybrid model is an effective method to support clinical decision making and artificial intelligence methods have great application potential in obstetrical practice. Future studies should also solve the problem of predicting the EDD within the scope of preterm delivery.

## Data Availability

The data that support the findings of this study are available from Hangzhou Women’s Hospital, but restrictions apply to the availability of these data, which were used under license for the current study, and so are not publicly available. Data are however available from the authors upon reasonable request and with permission of Hangzhou Women’s Hospital.
